# Porosity Effects on the Composite Girder by Rheological Dynamics and FEM

**DOI:** 10.3390/ma17235779

**Published:** 2024-11-25

**Authors:** Nataša Mrđa Bošnjak, Dragan D. Milašinović, Danica Goleš, Jelena Gučević, Arpad Čeh

**Affiliations:** 1 Faculty of Architecture, Civil Engineering and Geodesy, Univerisity of Banja Luka, University City, Bulevar vojvode Petra Bojovića 1A, 78000 Banja Luka, Bosnia and Herzegovina; 2 Faculty of Civil Engineering Subotica, Univerisity of Novi Sad, Kozaračka 2a, 24000 Subotica, Serbia; ddmil@gf.uns.ac.rs (D.D.M.); dgoles@gf.uns.ac.rs (D.G.); jgucevic@gf.uns.ac.rs (J.G.); ceh@gf.uns.ac.rs (A.Č.)

**Keywords:** porosity, RDT model, creep effects, concrete, FEM

## Abstract

A theoretical model for porous viscoelastoplastic (VEP) materials in the dry state is investigated in this research study. The model is based on the principles of conservation of mass and energy using the rheological dynamic theory (RDT). The model provides expressions for the creep coefficient, Poisson’s ratio, modulus of elasticity, damage variable, and strength as a function of porosity and/or void volume fraction (VVF). The reliability of the proposed model was analyzed by comparing numerical results with experimental ones on hardened concrete. A numerical model was created and analyzed in the commercial software Abaqus and validated by comparison with experimental data obtained by geodetic measurements on a composite wood–lightweight concrete girder. The deflections and stresses of the beam resulting from the influence of concrete creep and porosity were analyzed at the initial moment of time and after 6 years. The results showed that the RDT provided a reliable model for estimating parameters after exposure to long-term loads.

## 1. Introduction

Concrete used in civil engineering frequently cracks due to the initiation, growth, and coalescence of voids. Voids typically form around inclusions from particle cracking or decohesion at the interface between particles and the adjacent matrix. Environmental factors such as load, elevated temperatures, freeze–thaw cycles, and aggressive media alter the pore size, arrangement, and total porosity of hardened concrete structures. This situation pertains to the void volume fraction (VVF). Consequently, understanding the relationship between porosity and the mechanical properties of different concretes has become a crucial research area.

This paper examines a new mathematical-physical approach created as a result of previous research in the field of rheological dynamics [1,2,3]. Rheological dynamics combines dynamics and rheology to determine the response of a specimen or structure. Given that cracks and cavities in concrete structures change over time, degradation of mechanical characteristics occurs due to the opening and closing of cracks, especially if the structure or sample is exposed to cycles of freezing and thawing, elevated temperatures, etc. Observing the porosity as pores and cracks together, defined as the void volume fraction (VVF), rheological dynamics provides relationships between the VVF and mechanical characteristics by measuring, with non-destructive methods—only P and S wave velocities—because it is based on the propagation of elastic waves. In rheological dynamics, the relationships between Poisson’s ratio, creep coefficient, modulus of elasticity, damage variable, and stress depending on porosity are derived. Given that these are parameters related to long-term loads, rheological dynamics, based entirely on theoretical foundations, is a suitable basis for obtaining results regarding long-term loads from purely theoretical foundations.

In this paper, the derived relations between concrete porosity and its mechanical characteristics are first analyzed based on the experimentally obtained parameters of five concrete mixes. Then, the given connections are applied for the numerical analysis of a long-term loaded composite beam to determine if the proposed model implemented through the finite element numerical model gives the results obtained by measurement. Porosity, fundamentally tied to density variations, led to the defining of limit porosity values using strain energy density. Based on these values, the model parameters were established—the creep coefficient, Poisson’s ratio, damage variable, modulus of elasticity, and strength—as functions of porosity.

Numerous studies have utilized ultrasonic methods to estimate concrete parameters. For example, Ridengaoqier et al. [4] outlined experimental results for determining the porosity assessment methods of permeable concrete using ultrasonic testing. They investigated the impact of sample thickness on ultrasonic wave speed via the surface method, concluding that porosity can be estimated independently of sample thickness using this method. Additionally, the authors concluded that the relationship between porosity and ultrasonic wave velocity can be estimated using a quadratic function. A linear correlation between the cube root of the void fraction and the simulated compressive strength was proposed by Erkkilä et al. [5]. In this study, simulations were used to examine how porosity impacts the compressive strength of a quasi-brittle material. By analyzing existing expressions for the relationship between strength and porosity using the simulation data, a straightforward model was developed. In [6], Nithurshan and Elakneswaran offer a thorough analysis of the existing literature on models for predicting compressive strength, focusing on the accuracy and prediction mechanisms. They investigated various approaches, such as machine learning techniques. The review presented in their paper highlights the necessity for a foundational model that accurately forecasts the mechanical properties of concrete, encompassing aspects of physics, chemistry, and thermodynamics. In [7], Park, Ahmed, and Voyiadjis presented extensive theoretical and numerical research from recent decades on modeling concrete damage and plasticity with the aim of predicting concrete behavior using various approaches, including continuum damage mechanics.

Composite structures assembled from concrete and wood have been investigated by a number of authors, mostly experimentally and related to long-term loading. In this type of structure, the change in stress and deformation over time is very important.

Various problems have been analyzed in the literature for this type of structure. In [8], Fu et al. examined the connections between precast concrete and wood in a wood–concrete composite system bonded with epoxy resin. They altered parameters such as the types of concrete and wood, as well as the concrete surface treatment type, in order to analyze the mode of failure and the shear strength of the connections. In their study [9], Appavuravther, Vandoren, and Henriques explored the use of lightweight concrete in conjunction with glued laminated wood connected through a screw joint. In their study, Fu, Yan, and Kasal [10] provided the details of a four-point bending test carried out on wood–concrete composite beams. They observed the failure modes of both materials and offered analytical predictions of the effective bending stiffness, validated through experiments. In [11], Shi, Liu, and Yang examined the impact of long-term loads and experimentally derived the results from three wood–concrete composite beams. The samples were under continuous loading in a closed, uncontrolled environment. Mid-span beam deflections were monitored, and an evaluation of the deflection at the end of a 50-year service life was conducted. Another study that considered the long-term load was conducted by Tannert et al. [12], which involved experimentally testing two wood–concrete beams joined with adhesive and subjected to loads for approximately 4.5 years in an indoor environment. After this period, the beams were assessed for fractures, revealing that long-term loading did not degrade the adhesive bond. An overview of the literature implies that developing methods based on theoretical foundations is necessary for obtaining more accurate results.

The primary goal of this paper is to investigate a method that predicts more accurately the displacements and stress state of a composite girder, considering porosity as a time-dependent function dependent on the stress state of construction for long-term loads. This method provides accurately defined mechanical properties and shows that porosity defined through strain energy densities positively corrects the solution obtained for long-term loads.

The theory derived from rheological dynamics is applied in this paper to experimental data obtained from concrete cylinders in order to compare them with the results obtained by traditional methods. The application of defined expressions for the porosity obtained from energy densities in previous research was analyzed on samples but not on the structure itself. Due to the connection between existing stresses and porosity, that is, VVF, which, due to given stresses at a certain place in the structure, changes over time, a new modulus of elasticity was defined for the part of the structure based on the dependence of the modulus of elasticity and porosity derived according to RDT. Furthermore, it is proposed to apply a given elasticity modulus calculated for each part of the structure (e.g., finite element) depending on its VVF to obtain a certain parameter from the stress–strain state of the structure due to porosity. In the example analyzed in this paper, the parameter that is analyzed is the deflection in the middle of the composite beam, and the calculated deflections and the deflections obtained by measurement are compared to each other.

## 2. Materials and Methods (Porosity Analysis by Rheological Dynamics)

Compared to numerous models for analyzing the behavior of total wave propagation in inhomogeneous media with changes in density, the RDT provides a more comprehensive interpretation of the problem. In previous research, many expressions have been derived by a number of authors that relate mechanical properties to porosity. Many of these are unclear and cannot be applied to real materials. The model derived using the RDT is defined through the strain energy densities, on the basis of which porosities are obtained.

### 2.1. Model Parameters Description

Based on Bernoulli’s energy theorem, the relationship between Poisson’s ratio and the creep coefficient is derived in [1]:

(1) $$\varphi =\frac{2\mu }{1-2\mu }.$$

where φ and μ are, respectively, the creep coefficient and Poisson’s ratio.

The creep coefficient is also presented as a ratio of two moduli: the elastic modulus $E_{H}$, and viscoelastic modulus $E_{K}$:

(2) $$\varphi =\frac{E_{H}}{E_{K}}.$$

The linear dependence of the porosity and creep coefficient, according to [3], is presented in the following form:

(3) $$\varphi \left(p\right)=\varphi_{1}-\frac{p\left(\varphi_{1}-\varphi_{E}\right)}{p_{E}},$$

where $\varphi_{1}$ and $\varphi_{E}$ are, respectively, the creep coefficient defined at zero porosity and the creep coefficient at the end of the range of measurable porosities interval [0, $p_{E}$]. $p_{E}$ is the porosity defined at the end of measurable porosity. $p_{E}$ is the effective porosity, which indicates the ultimate porosity that can be measured by instruments.

If the linear relationship between the creep coefficient and the modulus of elasticity $E_{H}$ is taken into account, and according to the principle of conservation of mass states that the VE modulus $E_{K}$ is independent of porosity, Equation (2) implies a relationship between the modulus of elasticity $E_{H}$ and porosity p  of a linear type [3].

Considering Equations (1) and (3), the relationship between the porosity and Poisson’s ratio for the interval [0, $p_{E}$] is

(4) $$\mu_{RDT}\left(p\right)=\frac{\left[\varphi_{1}-\frac{p\left(\varphi_{1}-\varphi_{E}\right)}{p_{E}}\right]}{\left\{2\left[1+\varphi_{1}-\frac{p\left(\varphi_{1}-\varphi_{E}\right)}{p_{E}}\right]\right\}}.$$

where $\mu_{RDT}\left(p\right)$ is Poisson’s ratio as a function of porosity.

According to [1], the scalar damage variable D of VEP material for the interval [0, $p_{E}$] is

(5) $$\frac{\varphi_{1}}{1+\varphi_{1}}\geq D\geq \frac{\varphi_{E}}{1+\varphi_{E}},$$

where the initial (critical, according to Lemaitre [13]) damage variable $D_{1}$ at zero porosity corresponds to the creep coefficient $\varphi_{1}$. Consequently, the nonlinear relationship between the damage variable and porosity is

(6) $$D\left(p\right)=\frac{\left[\varphi_{1}-\frac{p\left(\varphi_{1}-\varphi_{E}\right)}{p_{E}}\right]}{\left\{1+\left[\varphi_{1}-\frac{p\left(\varphi_{1}-\varphi_{E}\right)}{p_{E}}\right]\right\}}.$$

### 2.2. Void Volume Fraction

According to [3], an expression for Poisson’s ratio depending on the elastic moduli ratio Ψ is

(7) $$\mu_{1/2}=\frac{\left(\psi -1\right)\pm \sqrt{\psi^{2}-10\psi +9}}{4},$$

where Ψ is directly proportional to the modulus of elasticity $E_{H}$ and inversely proportional to the dynamic modulus of elasticity $E_{D}$:

(8) $$\Psi =\frac{E_{H}}{E_{D}}.$$

Based on two Poisson’s ratios obtained, the two stress–strain curves are [3]:

(9) $$\sigma_{i}=\frac{1}{2K_{E,i}}\left(\sqrt{1+4K_{E,i}E_{i}\left(0\right)\varepsilon }-1\right),\;\;\;\;\;i=1,2,$$

where $K_{E,i}$ and $E_{i}\left(0\right)$ are, respectively, structural—material constants and tangent modulus (modulus of the material in the initial state) defined as follows:

(10) $$K_{E,i}=\frac{\varphi_{i}}{\sigma_{ef}},\;\;\;\;\;\varphi_{i}=\frac{2\mu_{i}}{1-2\mu_{i}},\;\;\;\;\;E_{i}\left(0\right)=E_{H}\left(1+\varphi_{i}\right),\;\;\;\;\;i=1,2,$$

According to Murakami [14], the effective stress $\sigma_{ef}$ includes the effects of stress concentration in voids and the effects of interaction between voids, in addition to the effect of the reduction of the geometric cross-section area due to damage, and is represented by the expression

(11) $$\sigma_{ef}=\frac{f_{cS}}{1-D_{1}},$$

where $f_{cS}$ is the uniaxial unconfined compressive strength (UCS) of the damaged dry sample.

The damaged state of VEP materials can be described by the VVF [14,15,16]. The VVF is the total porosity represented by

(12) $$VVF=\frac{dV-dV_{0}}{dV}.$$

The volume of a representative volume element (RVE) at a specific point in a material is denoted by dV, whereas $dV_{0}$ represents the volume of the matrix within the RVE. According to Goods and Brown [17], voids begin to merge when they expand to a size comparable to the distance separating them, and at that stage, the VVF ranges from 0.15 to 0.25 (Needleman and Tvergaard [18]).

While Equation (12) defines the total porosity, measuring VVF directly is challenging. Contrarily, measuring the change in material density becomes relatively straightforward, and the total porosity can be calculated according to

(13) $$VVF=1-\frac{\rho }{\rho_{0}},$$

where the damage density and the initial density of the material are represented by ρ and $\rho_{0}$, respectively.

Lemaitre and Dufailly [19] utilized the variation in density between the initial and damaged states to interpret the micromechanical model of a spherical void in a spherical representative volume element, demonstrating the connection between the damage variable and porosity

(14) $$D_{LD}={\left(1-\frac{\rho }{\rho_{0}}\right)}^{\frac{2}{3}}.$$

This relation describes the connection between surface damage and the changes in density or porosity.

### 2.3. Strain Energy Densities

In [3], Milašinović gave a detailed explanation of the limit values of VVF based on the strain energy densities. The VVF must be determined when the specimen is under load. The experiments described by Wu et al. [20] and Yang et al. [21], which were analyzed to determine the porosity and permeability of porous concrete, are suitable for this. The experiments from the papers [20,21] are connected and analyzed according to the RDT in the paper [22].

Considering that under isothermal and adiabatic conditions, only a portion of the work done by the external load is stored as elastic energy density, the remaining work is used for the development of inelastic deformations and cracks, referred to as energy density dissipation. This dissipation results from the differences in energy densities caused by the positive and negative values of Poisson’s ratios used for obtaining the corresponding stress–strain curves. The strain energy density in the ideal elasticity range $W_{el}$, strain energy density obtained from the stress–strain curve for the positive value of Poisson’s ratio $W_{d1}$, and strain energy density obtained from the stress–strain curve for the negative value of Poisson’s ratio $W_{d2}$ are:

(15) $$W_{el}=\frac{1}{2E_{H}}\sigma_{ef}^{2},$$

(16) $$W_{d1}=\int_{0}^{\varepsilon_{cF}}\sigma_{1}d\varepsilon =\int_{0}^{\varepsilon_{cF}}\frac{1}{2K_{E1}}\left(\sqrt{1+4K_{E1}E_{1}\left(0\right)\varepsilon }-1\right)d\varepsilon =\frac{1}{2K_{E1}}\left[-\varepsilon_{cF}+\frac{-1+{\left(1+4K_{E1}E_{1}\left(0\right)\varepsilon_{cF}\right)}^{\frac{3}{2}}}{6K_{E1}E_{1}\left(0\right)}\right],$$

(17) $$W_{d2}=\int_{0}^{\varepsilon_{cF}}\sigma_{2}d\varepsilon =\int_{0}^{\varepsilon_{cF}}\frac{1}{2K_{E2}}\left(\sqrt{1+4K_{E2}E_{2}\left(0\right)\varepsilon }-1\right)d\varepsilon =\frac{1}{2K_{E2}}\left[-\varepsilon_{cF}+\frac{-1+{\left(1+4K_{E2}E_{2}\left(0\right)\varepsilon_{cF}\right)}^{\frac{3}{2}}}{6K_{E2}E_{2}\left(0\right)}\right].$$

According to [3], the total energy density dissipation is:

(18) $$W_{d}=W_{d1}-W_{d2}.$$

As a result, the total porosity derived from the energy densities, denoted as $p_{max}$, can be calculated in the following manner:

(19) $$p_{max}=\frac{W_{d}}{W_{el}}=\frac{W_{d1}-W_{d2}}{W_{el}}.$$

As a positive Poisson’s ratio can be measured, whereas a negative one cannot, this fact enables the establishment of the boundary of measurable VVF:

(20) $$p_{E}=\frac{W_{d1}-W_{el}}{W_{el}}.$$

By considering the difference between the $W_{d1}$ and $W_{d2}$ in relation to the $W_{d}$, the VVF controls the macroscopic failure of the sample by the static strength $f_{US2}$; that is, the effective porosity corresponding to the fracture of the sample $p_{f}$ [3] is:

(21) $$p_{f}=\frac{2W_{el}-W_{d1}-W_{d2}}{W_{d}}.$$

The numerator of the fraction in relation (21) divided by $W_{el}$ determines the minimum VVF; that is, the porosity of the sample obtained by non-destructive methods when the sample is loaded only due to its weight,$p_{min}$.This porosity governs the dynamic strength $f_{US1}$:

(22) $$p_{min}=\frac{2W_{el}-W_{d1}-W_{d2}}{W_{el}}.$$

### 2.4. Relationship Between Stress and VVF

In [1], the relationship between strength and the stress at the limit of elasticity is determined as

(23) $$\frac{\sigma_{E}}{f_{cS}}=\frac{\varphi_{1}}{1+\varphi_{1}}=D_{1}.$$

Numerous studies indicate that the progression of damage or strength degradation in concrete does not follow a linear pattern [23,24]. Thus, the nonlinear correlation between porosity and the strength of dry VEP material $\sigma_{f}$, according to the RDT as is derived in [3], is

(24) $$\sigma_{f}=\sigma_{ef}\left\{1-\frac{p_{f}}{p_{E}}\left[1-D_{1}\left(1-D_{f}\right)\right]\right\},$$

where $p_{E}$ is the porosity at the end of the measurable porosity range, while $D_{f}$ is a porosity-dependent damage variable.

### 2.5. Finite Element Porosity

To obtain the porosity from the known stress in the limit state (t), two roots were derived in the following form:

(25) $$p=\frac{-B-\sqrt{B^{2}-4AC}}{2A},$$

where

(26) $$A=\varphi_{1},$$

(27) $$B=\varphi_{1}p_{E}\left(\frac{\sigma_{y}}{\sigma_{ef}}-1\right)-p_{max}\left(1+\varphi_{1}-D_{1}\right),$$

(28) $$C=p_{max}\left(1+\varphi_{1}\right)p_{E}\left(1-\frac{\sigma_{y}}{\sigma_{ef}}\right).$$

where $\sigma_{y}$ is the known stress obtained from the finite element analysis in time t.

This method allows for the calculation of the time-dependent material properties for all porous finite elements within the discretized FEM mesh.

Based on the obtained porosity, the modulus of elasticity can be calculated based on the linear dependence of the modulus of elasticity and porosity:

(29) $$E_{H,fe}\left(p\right)=E_{H0,fe}\left(1-\frac{p_{fe}}{p_{\;max,fe}}\right).$$

Using the adjusted modulus of elasticity, the corrected influences on the girder can be calculated, thus accounting for the impact of porosity under long-term loading.

**Figure 1:**
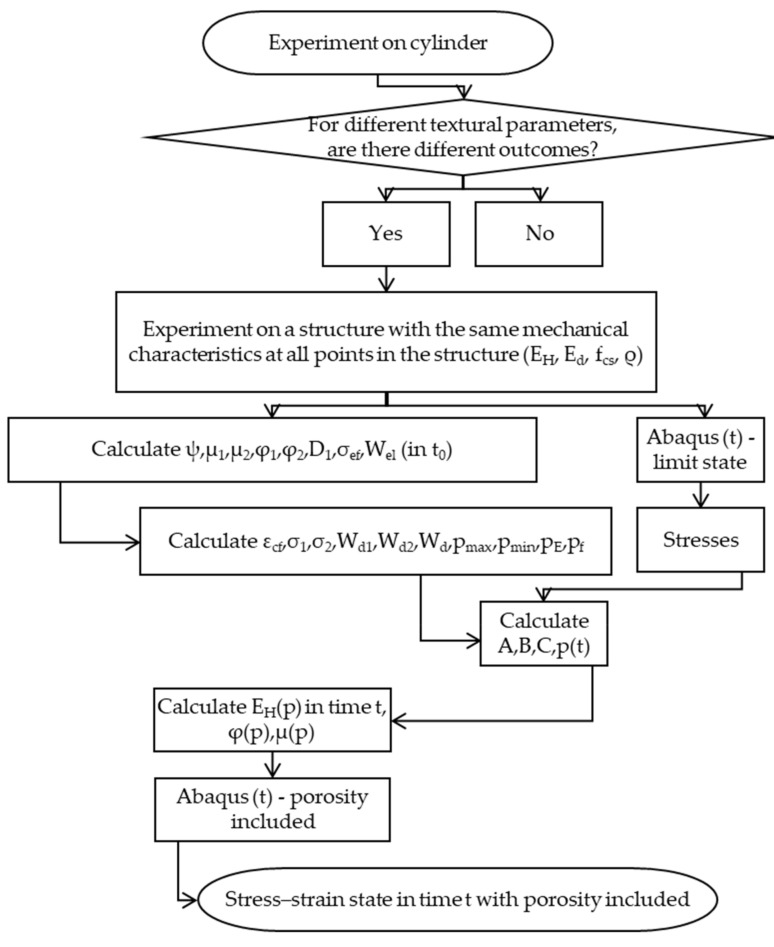
RDT method implementation in FEM analysis.

A flowchart for the described method of implementation is presented in Figure 1.

## 3. Experimental and Numerical Results

Quasi-static stress–strain curves, according to the RDT, can be calculated when the compressive strength, Poisson’s ratio, elastic modulus, and concrete density are determined through experimental methods. The author in [1] described this procedure using the concrete cylinder SG, which was cast in standard cylindrical molds (300 mm height and 150 mm diameter) and tested at the standard age of 28 days. The experimental research was conducted at the Materials and Structures Testing Laboratory of the Faculty of Civil Engineering in Subotica.

This paper demonstrates that the proposed RDT model distinctly highlights variations in the response of concrete cylinders when assessing the four material properties using traditional methods. These methods and their connection to the RDT are described in detail in [1,2].

The second part of the experimental work was also conducted at the Materials and Structures Testing Laboratory of the Faculty of Civil Engineering in Subotica and aimed to demonstrate the effect of porosity on a concrete–wood composite beam during long-term loading, according to the RDT model. The experimental data were measured at the initial moment of time ($t_{0}$), i.e., at the moment after placing the beam on the supports, which was done 28 days after pouring the concrete slab on the wooden beam. The second measurement, which was carried out by geodetic measurements, was conducted after 6 years (time t), and the parameter values obtained on the model created in the commercial software package Abaqus were compared with the given measurements. The following chapters present the details of the experimental and numerical analyses with a critical review of the results obtained.

### 3.1. Experimental Results

In the first part of the paper, four types of concrete mixes were examined. The measured and computed parameters according to the RDT model for all four types of concrete mixes are shown in Table 1. Each sample was observed separately, and the measurements were made with precision in accordance with the standards. The introduction of statistical methods that would be based on a large number of concrete samples of the same or similar composition was not done in this paper. The calibration of the model parameters is based on the measured velocities of the P and S waves on standard concrete samples, and the effect of porosity is included through the functional dependence of parameters in relation to the porosity.

SG-type concrete is a low-shrinkage, expanding material (SikaGrout^®^-212, manufactured by Sika Serbia, located in Pećinci, Serbia) comprising a powdered mix of cement, powdered cement additives, and crushed stone aggregate. This concrete is not standard, possessing relatively high strength and high deformability compared to other concrete types. The SG1 and SG2 cylinders were made from the same expanding material as the SG cylinder but with the water/solid ratio reduced (0.12). This aimed to achieve lower porosity, higher density, and higher strength. The SG3 and SG4 cylinders included an air-entraining agent (1% dosage by weight of solid content, Sika Lightcrete^®^-02, manufactured by Sika Serbia, located in Pećinci, Serbia) to reduce their density and therefore increase porosity. Experience indicates that this mixture alteration results in a lower cylinder strength (Table 1).

**Figure 2:**
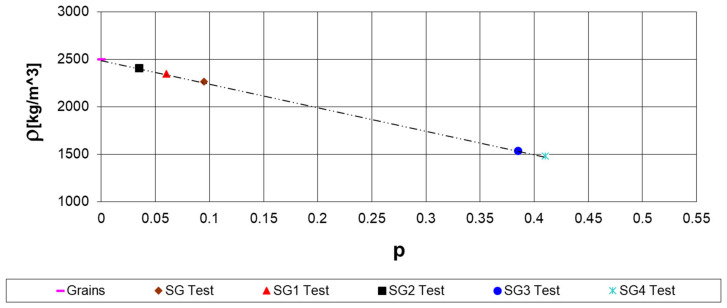
Tested porosities versus concrete densities.

Figure 2 illustrates the relationship between concrete densities and the corresponding tested porosities.

**Figure 3:**
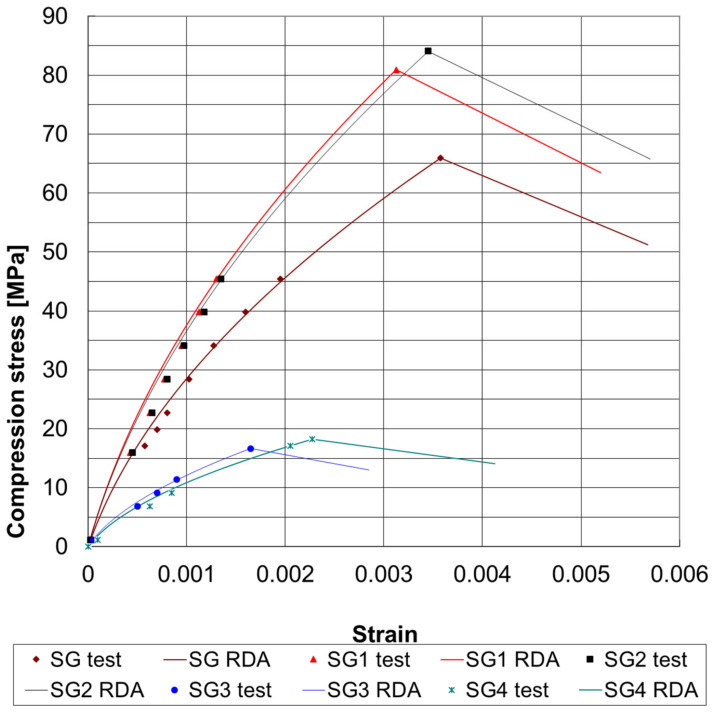
Quasi-static stress–strain curves, determined by rheological dynamical analysis (RDA) modeling, for the SG concretes.

Figure 3 illustrates the quasi-static stress–strain curves, both experimental and calculated, for the SG concretes. This research, as used in [2], demonstrates that the model effectively highlights the differences in concrete cylinder responses determined by standard testing methods.

**Figure 4:**
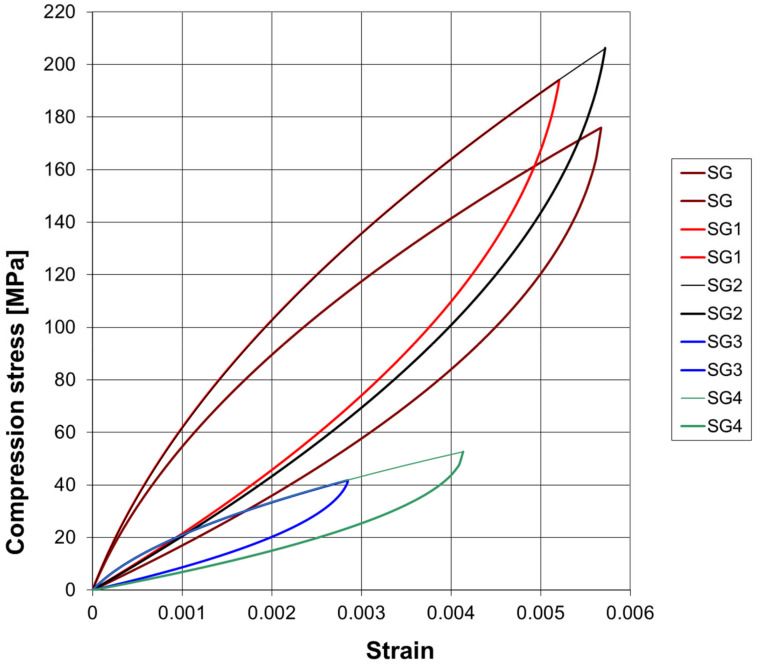
Dynamic stress–strain curves for the SG concretes determined by RDT modeling.

Table 2 presents the model’s calculated parameters for both dynamic curves of the SG concrete cylinder, as shown in Figure 4. The static strength of 56.36 MPa is below the tested compressive strength, which is 65.88 MPa. This clearly indicates softening behavior.

According to [3], strain energy densities, limited VVSs, and damage variables are calculated for all five types of concretes (Table 3):

The energy densities are evidently higher for the samples with greater compressive strength.

**Figure 5:**
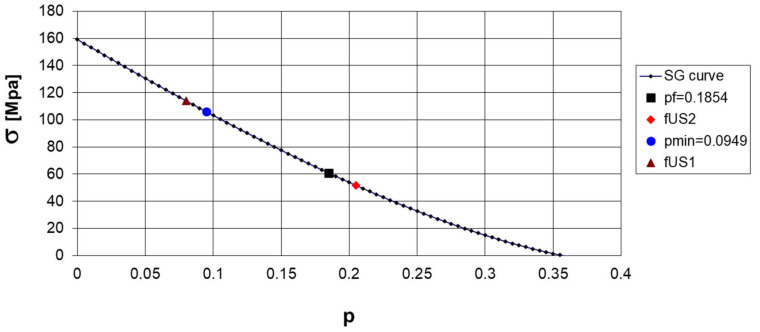
Porosity as a function of compressive strength for the SG concrete.

If we examine how the porosity influences the peak damage variable and, according to the damage variable calculated, how it influences the stress, we can see that the experimentally determined compressive strength closely matches the strength of the cylinder with a confirmed fracture porosity. Figure 5 illustrates how the strength varies with porosity.

The model demonstrates that static strength depends on a porosity that controls for macroscopic failure, whereas dynamic strength relies on a minimum porosity. This result makes sense, as dynamic loading occurs quickly under small loads and density changes.

**Figure 6:**
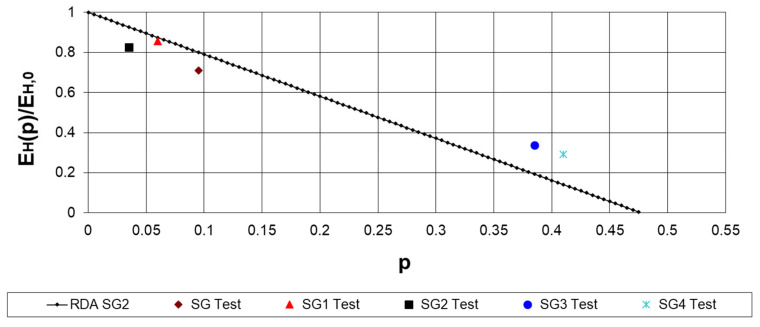
Tested dimensionless Young’s modulus as a function of porosity in comparison with the straight line for the SG2 concrete.

Figure 6 compares the tested and calculated dimensionless elasticity moduli. The elasticity modulus at zero porosity is defined according to [3] for the SG2 cylinder, which possesses the highest compressive strength.

Table 4 indicates that the tested strengths align well with the calculated values.

**Figure 7:**
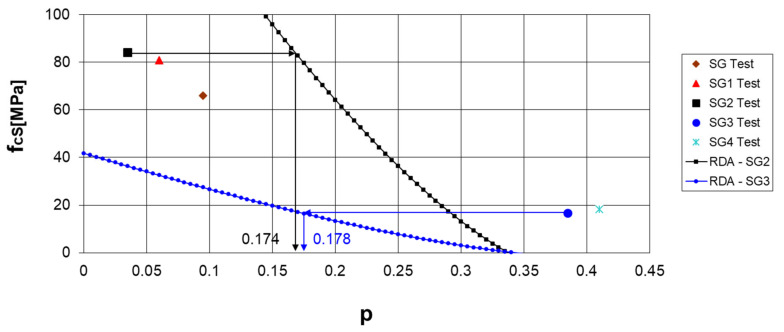
Tested compressive strengths compared with the curves obtained by RDT modeling for the SG2 and SG3 concretes.

Figure 7 illustrates the comparison between tested compressive strengths and RDT curves for SG2 and SG3 concrete cylinders. Despite cylinder SG2 having an experimental porosity of 0.0368 and SG3 cylinder having a porosity of 0.3847, both cylinders at failure exhibit nearly the same critical porosity of 0.17 and a critical damage variable of 0.48.

Figure 7 also illustrates two distinct physical processes. When the measured porosity is less than calculated (indicated by the black line), additional voids appear as cracks due to load until failure, forming the final VVF. Conversely, when the measured porosity exceeds the calculated value, the load affects close parts of the voids (indicated by the blue line).

### 3.2. Numerical Finite Element Analysis of Porous Composite Beam

**Figure 8:**
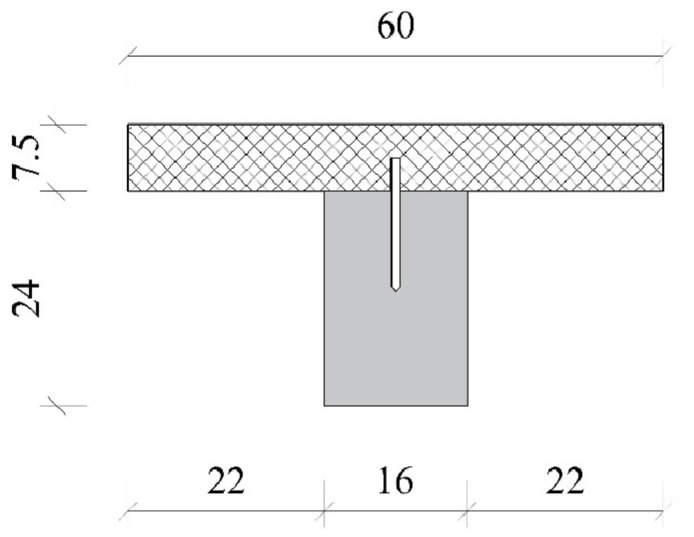
Cross-section of a concrete–wood beam (cm).

**Figure 9:**
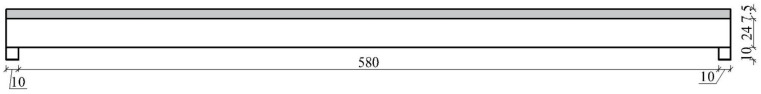
Longitudinal section of a concrete–wood beam (cm).

The composite girder under consideration, with a T cross-section (Figure 8), consists of a flange made of lightweight concrete and a web made of monolithic wood. The tested girder is six meters long, with the distance between the supports being 5.8 m (Figure 9). To transfer the shear forces from the concrete slab to the wooden beam, steel screws for wood without nuts were used (Ø10/150), which were placed at a distance of 20 cm apart. Experimental research on the girder from this example, exposed to long-term environmental actions, was carried out at the Faculty of Civil Engineering in Subotica. Tests were performed first at the initial time (28 days after pouring concrete) and again after six years of exposure to external actions. Over six years, the structure was positioned in front of the faculty building, which shielded it from extreme environmental events.

The mechanical characteristics of the wood and concrete obtained at the initial moment and, according to the rheological dynamic theory, at time t are given in Table 5.

**Figure 10:**
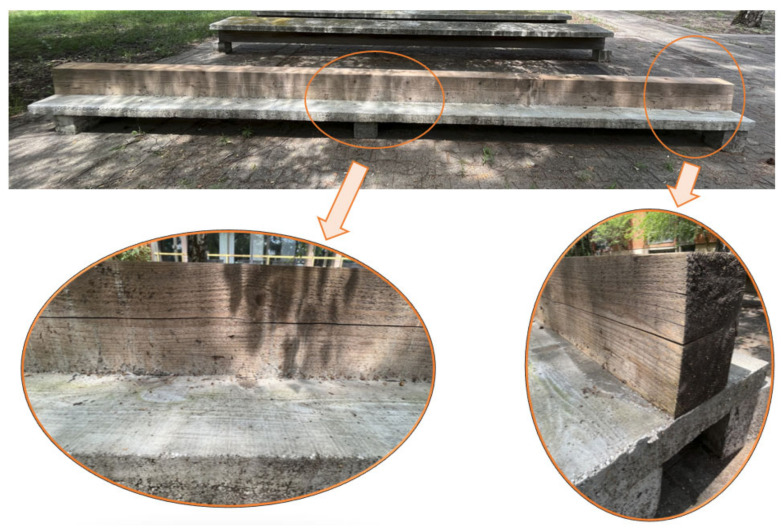
Concrete–wood beam after 6 years of exposure to atmospheric influences.

The deflection at the middle of the span, measured at the initial moment (after 28 days), was 1.74 mm. After the initial measurements, the beam was exposed to the influence of the environment to examine the concrete porosity’s influence on the behavior of the girder. After six years, the wooden beam and concrete flange reached a new condition, where a longitudinal crack appeared on the entire length of the wooden beam, positioned 10 cm above the bottom fiber (Figure 10).

Considering that for the preserved part of the composite beam above the longitudinal crack, in the limit state, the hypothesis of strain equivalence must be valid, and the value of Poisson’s ratio of the concrete slab, μ=0.35, is adopted for both materials.

The concrete–wood composite girder was modeled in the Abaqus software package, and the model was validated by comparison with the experimental results. A 3D model was created, and the variants of the coupling of the concrete slab with the wooden beam were analyzed at the initial time and after six years.

To provide the most valid representation of the real model on which the experimental measurements were made, a girder with screws and a rigid connection over the entire surface between the concrete plate and the wooden beam was modeled at time $t_{0}$, and time t = 6 years. The embedded region category of constraints from the Abaqus library was used for the screws-to-concrete and screws-to-timber constraints. For the simulation of the rigid connection of the beam and the plate, the tie constraint category of constraints was used, which represents the two fully connected parts of the girder. Convergence was achieved with a mesh size of 0.015 [m]. A total of 154,250 elements were generated for the given mesh, of which 152,400 were linear hexahedral elements of type C3D8, with which the concrete slab and wooden beam were modeled, and 1830 were linear quadrilateral elements of type R3D4, along with 20 linear triangular elements of type R3D3 from the group of elements for modeling three-dimensional rigid bodies, which were used to model the supports.

The screws significantly affected the behavior of the girder. Before the appearance of the longitudinal crack, they provided the composite action of concrete and wood. After six years and the appearance of a longitudinal crack, the screws lost their function and were therefore excluded from the analysis.

#### 3.2.1. Model of Girder with Screws at the Initial Moment of Time $t_{0}$

**Figure 11:**
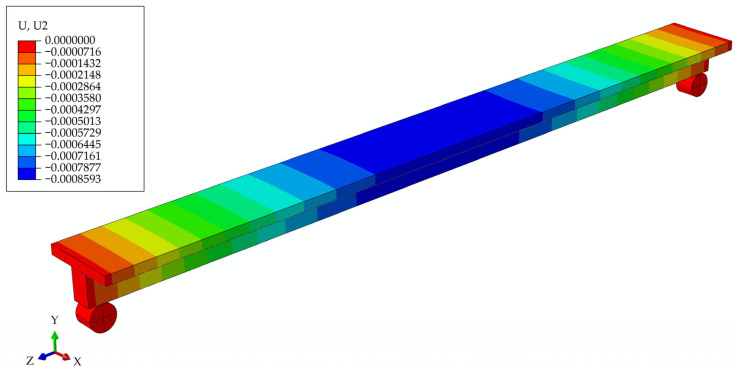
Beam deflections at the initial moment of time $t_{0}$ for the model of girder with screws.

In Figure 11, the deformed girder is presented at the initial moment, with the deflection values in the legend.

#### 3.2.2. Model of Girder with Screws at the Moment of Time t = 6 Years

**Figure 12:**
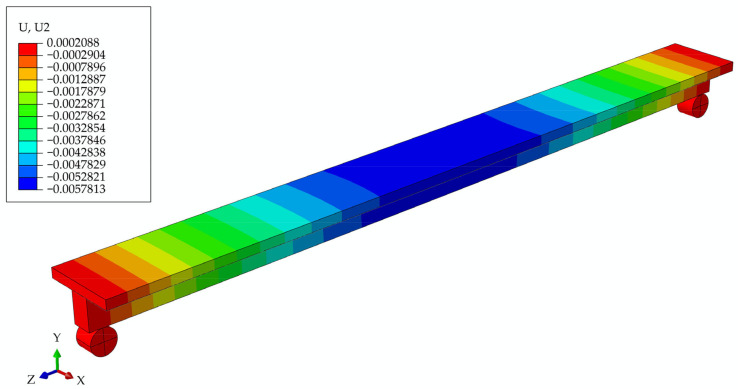
Beam deflections at t = 6 years for the model of a girder with screws.

**Figure 13:**
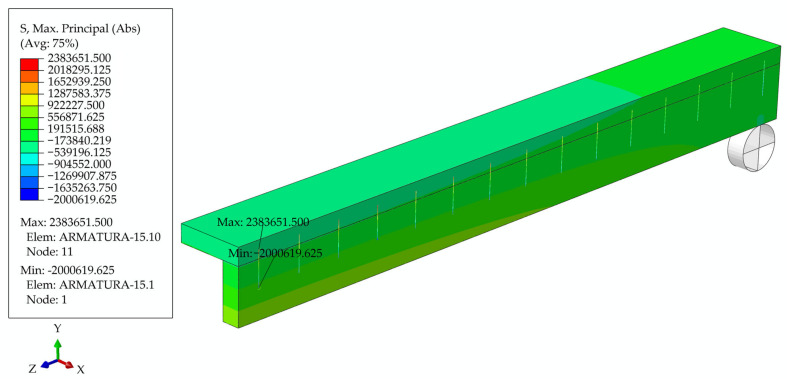
Longitudinal and transverse cross-section through the middle of the girder with the distribution of maximum stress at the moment t = 6 years.

In Figure 12, the deformed girder is presented at t = 6 years, with the deflection values in the legend, and, in Figure 13, the longitudinal and transverse cross-section through the middle of the girder with the distribution of maximum stresses.

The change in stress did not occur due to a change in the load but was due to the effect of the creep of concrete on the screws, as happens with the prestressing force in prestressed structures. This stress change caused a shear stress in the beam 10 cm above the bottom of the wooden beam. At the top of the screws that connected the wood, the maximum stress value is 2 [MPa], and this exceeds the tensile strength of the wood perpendicular to the fibers, which is, according to [25], 1.7 [MPa].

#### 3.2.3. Model of Girder Without Screws at the Initial Moment of Time $t_{0}$

**Figure 14:**
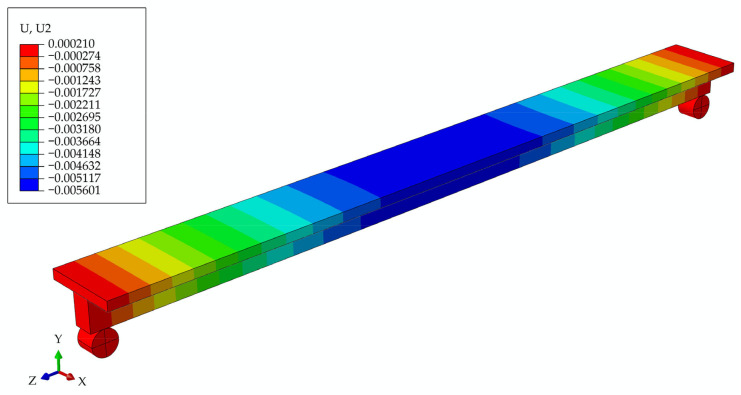
Beam deflections at the initial moment of time t_0_ for the model of the girder with only frictional connection.

The previously analyzed model is also examined without screws, with only a frictional connection between the concrete slab and the wooden beam (Figure 14).

#### 3.2.4. Model of the Girder Without Screws at t = 6 Years

**Figure 15:**
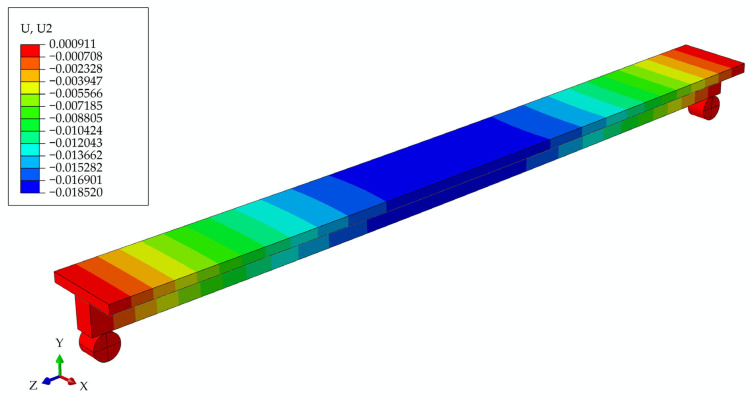
Beam deflections at t = 6 years for the model of girder with only frictional connection.

For the observed case, a model was analyzed in which, due to the crack, the screws are omitted because they no longer perform their function after 6 years (Figure 15); deflections and the stress-state change due to creep. The connection between the concrete slab and the wooden beam is achieved by friction.

The vertical displacement at the middle of the concrete slab is v=18.434 [mm].

#### 3.2.5. Model of Girder Without Screws at t = 6 Years with Porosity Influence

**Figure 16:**
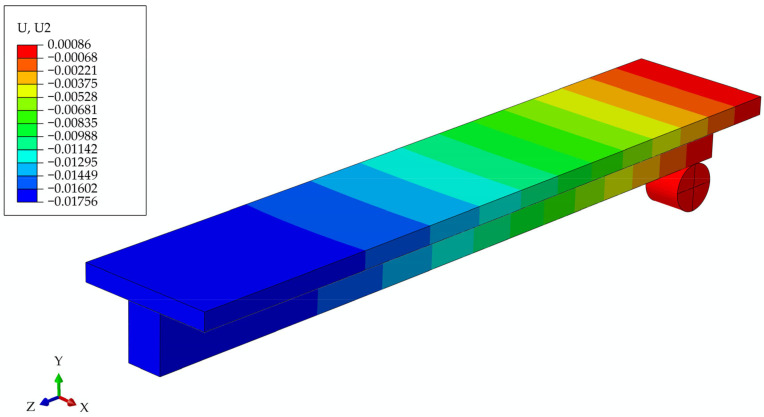
Beam deflections at t = 6 years for the model of the girder with only frictional connection and with porosity influence (half of the girder).

According to the derived expressions for the relationship between porosity and stresses, the porosity was calculated using stresses obtained due to the influence of creep, and the new corrected modulus of elasticity adopted for all elements of the concrete plate was also calculated. With this input, the resulting deflection due to the effect of porosity and creep at the middle of the span of the concrete slab is v=17.5 [mm] (Figure 16). This result was expected since it provides smaller deflections than those obtained solely due to the influence of creep.

#### 3.2.6. Comparison with Geodetic Measurements

**Figure 17:**
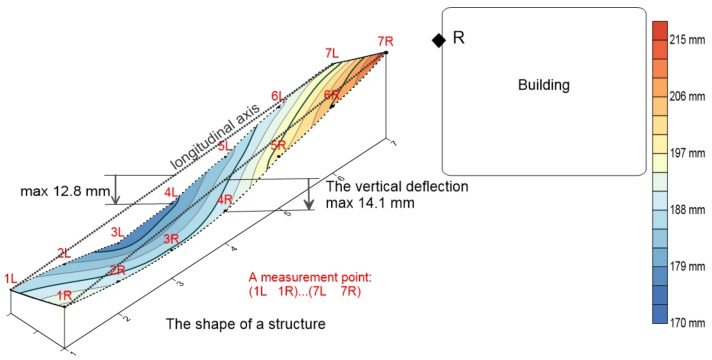
Measured geodetic profiles.

To measure the deflections after 6 years of exposure to atmospheric conditions, various equipment and accessories were assembled, including a Leica DNA03 Digital Level with two invar staffs, an umbrella, a tripod, the record, a sketch, and calculation tools. The instrument underwent testing and adjustment following the ISO 17123-2 procedure [26], achieving a standard deviation height measurement per 1 km and with invar staffs of 0.3mm/km [27]. The girder’s deflection was measured at selected points based on the structure’s profiles relative to the fixed point (point R) installed in the Faculty of Civil Engineering building in Subotica (Figure 17). The measurements were conducted to meet the necessary accuracy standards, with a line-of-sight ranging from 6 to 13 m (Table 6).

Data processing and the determination of the vertical deflections for seven profiles, each with two measuring points, involved calculating the point heights per profile, identifying differences, and comparing the resulting values [28]. Vertical deflections for each structure were calculated based on the benchmark height. Figure 17 displays the geodetically measured deflections at the midpoint of the concrete slab.

Comparing the measured deflections with the deflections obtained for the model, it can be seen that the results obtained for the model in Abaqus, considering only the creep effects, gave slightly higher deflections than the measured ones. This fact supports the claim that it is necessary to introduce the influence of porosity to obtain values closer to the measured ones. By introducing porosity in the model, as defined according to rheological dynamics, the results that were obtained are much closer to the measured ones than in the case where only the effect of creep is taken into account.

## 4. Discussion

This paper analyzes the new method presented in [3] for obtaining porosity through energy densities in the context of rheological dynamics. The essence of the proposed method for introducing the porosity effects is that porosity is related to the load on the structure because the stresses in the structure change under the load. By connecting this with energy considerations, this study establishes the functional dependencies of model parameters and porosity.

In the first part of this paper, the experimentally obtained results for the SG cylinders were compared with the parameter values obtained through the energy densities according to the proposed RDT model.

The analysis in this part of the paper showed that the proposed RDT model clearly emphasizes the differences in the response of concrete cylinders when the four material characteristics are evaluated by traditional methods. Detailed comparisons of the results obtained by the RDT with traditional methods are discussed in [1]. Also, two distinct physical processes are evident. When the measured porosity is less than the calculated value, additional voids manifest as cracks under load, leading to the formation of the final VVF at failure. In contrast, when the measured porosity exceeds the calculated value, the load effects result in the closure of parts of the voids.

To validate the concepts of porosity in rheological dynamics theory due to a long-term load, in the second part of this paper, the composite wood–lightweight concrete girder was analyzed after six years. Geodetic deflection measurements were taken, and the comparison between the measured results and those obtained numerically at the midpoint of the girder confirmed satisfactory agreement.

The coupled girder was analyzed under the influence of its weight, simulating the stress during exposure to atmospheric actions for 6 years. The deflection of the girder, in the middle of the span, at the initial moment of measurement (28 days after pouring the concrete) was 1.74 mm, while the measured deflection after 6 years for different positions of the same cross-section was between 12.8 and 14.1 mm.

The models of the girder in the Abaqus commercial software were made to simulate both a stress–strain state at the initial time and after 6 years. A 3D static analysis was conducted on a simply supported composite girder with a span of 5.8 m, a T cross-section consisting of a wooden rib with dimensions of 0.16*0.24 m, and a concrete flange with dimensions of 0.075*0.6 m. The material properties for the initial moment and the limit state (t = 6 years) are defined according to Table 5.

The screws were made of steel and were positioned every 0.2 m along the beam. The properties of the steel used for the screws included a Young’s modulus of 200 GPa, a Poisson’s ratio of 0.3, and a density of 7850 kg/m^3^. The screws were modeled as wire elements and were embedded in the surrounding materials using the embedded region type of constraint.

In all four models, the beam was subjected to its weight, and a gravity-type load was used. The supports were modeled as 3D discrete rigid parts, with surface-to-surface contact between the supports and the wooden beam. The convergence study was conducted on all four models, revealing that a consistent element size of 0.015 m across these models resulted in deflection convergence. Concrete slabs and wooden beams were modeled with linear hexahedral elements of type C3D8. Linear quadrilateral elements of type R3D4 and triangular elements of type R3D3 from the group of elements for modeling three-dimensional rigid bodies were used to model the supports. The type of analysis that was carried out for all models was static and general. Creep was introduced into the model through the parameters derived from the RDT, modulus of elasticity, and Poisson’s ratio.

The connection between concrete and wood was modeled in two ways. First, a rigid connection was created using the Tie constraint from the Abaqus palette. This approach simulated the connection between the concrete poured directly onto the wooden beam and the formwork. The second type of connection employed was a friction connection, which was used to simulate the failure of a rigid connection between the wood and concrete after 6 years. In the model where it is assumed that the screws and the rigid connection between concrete and wood remained unchanged over time, the deflection obtained at the initial moment was 0.859 mm. After 6 years, it reached the value of 5.781 mm. A model of a girder with the influence of creep, with the assumption that the screws and the rigid connection between concrete and wood lost their properties over time, gave the resulting deflection after 6 years of 18.434 mm.

Considering the impact of porosity on the model and calculating the parameters through energy densities, a smaller deflection was obtained than the deflection obtained only by calculating the influence of concrete creep. The deflection obtained when the porosity of the concrete slab was taken into account, calculated for all the finite elements with the maximum value of the stress taken from the concrete plate, was 17.5 mm. This indicates that the porosity effectively corrects the result obtained at time t, bringing it closer to the measured value.

This analysis applies to dry, porous concrete. Considering the unknowns related to open or closed pores (cracks) in concrete thus aids in understanding the critical VVF of natural or artificial materials with cracks and eventual disintegration. Still, it must include new information about material behavior, especially under natural conditions. The porosity limits derived using the RDT include total porosity, effective porosity, and minimum porosity. Proof of the boundary porosities is discussed in study [22], which is based on the experiments detailed in papers [20,21]. This proof can be enhanced by the measurements taken under specimen loads, which are similar to those conducted in permeability research.

Given that the porosity of a viscoelastic material, such as concrete, with a creep coefficient and an aging coefficient as a function of porosity, has not been investigated, no comparison with examples from the literature was possible.

The limitation, when comparing the measured results and the results obtained according to the RDT model, is certainly the fact that only the porosity of the concrete part of the composite beam was investigated, while for the wooden beam, only the creep effect was taken into account. For this reason, the authors have not yet investigated the porosity of the wood.

Considering that the model provides solutions with the porosity effects closely aligning with the measured values, further research could be directed toward applying this model to wood, potentially leading to more accurate results. Additionally, future investigations will focus on more precise laboratory measurements.

The model could include higher loads over the described parameters, which would then change. However, some extreme climates and exceptional dynamic loads could call into question the existing numerical model because the parameters of the model were not formulated for these conditions in the theoretical setting.

Investigations of porosity and permeability using the model were presented in paper [22] and applied to the experimental results from paper [20]. Research with dynamic loads is planned to confirm the experimental results that are currently confirmed for static loading.

## 5. Conclusions

The aim of this study was to investigate the application of the method developed by the rheological dynamic theory for long-term loads on a wood–concrete composite girder. The findings in this study showed that deflections due to long-term loading approach the experimental results if porosity is taken into account. Porosity is considered a critical textural parameter in damaged materials, which has a great influence on the stress state of the material, as shown in the first part of the paper. The numerical model showed that due to the change in porosity on the concrete part of the structure from 0.13 to 0.36 after 6 years, there was a change in deflections. More precisely, the deflection of 18.5 cm, obtained in the middle of the beam girder by taking into account the creep, was reduced to 17.5 cm by taking the porosity into account, while the experimentally obtained deflection was 14 cm. 

This study is limited by the investigation of only the concrete part of the structure, and a suggestion is given for the continuation of the investigation of the porosity of the wooden part of the structure in subsequent research. Parameterization in Abaqus can be performed using Python script, which is also a subject for further research. This would enable additional parametric studies. Applicability under dynamic loading conditions should also be investigated to confirm the experimental results currently confirmed for static loading. The main contribution of this paper is the verification of a new model for calculating stress–strain values due to the influence of porosity for long-term loaded structures. This approach, which combines the influence of creep and porosity, provides results closer to the experimental ones than if only creep was to be taken into account.

## Figures and Tables

**Table 1:** Tested and calculated mechanical properties for the quasi-static curves.

**Concrete**	ρ [kg/m^3^]	$p_{test}$	$E_{H}$ [MPa]	$\mu^{*}$	$f_{cS}$ [MPa]	$\varphi^{*}$	$K_{E}^{*}$	$\varphi_{1}$
SG	2265.5	0.0949	30994	0.185	**65.88**	0.5873	0.025344	1.67
SG1	2347	0.0623	37285	0.2	**80.9**	0.6666	0.017293	1.4
SG2	2411	**0.0368**	36031	0.198	**84.1**	0.6556	0.017243	1.45
SG3	1540	**0.3847**	14670	0.213	**16.6**	0.7422	0.091702	1.52
SG4	1480	0.4087	12725	0.225	**18.2**	0.8182	0.103829	1.89

**Table 2:** Model parameters for both dynamic curves of the SG concrete.

$\mu_{1}$	$\varphi_{1}$	$v_{T1}$ [m/s]	$\kappa_{1}$	$E_{1}\left(0\right)$ [MPa]	$K_{E1}$	$f_{US1}$ [MPa]
0.312766	1.67045	2282.697	0.272446	82767.94	0.009495	127.29
μ _**2**_	φ _**2**_	$v_{T}$_**2**_ **[m/s]**	κ _**2**_	E _**2**_ **(0) [MPa]**	$K_{E}$ _**2**_	$f_{US}$_**2**_ **[MPa]**
−0.455108	−0.476499	3543.125	0.656383	16225.39	−0.002708	56.36

**Table 3:** Energy densities, VVFs, and damage variables for five types of SG concretes.

**Concrete**	$W_{el}$ [MJ/m^3^]	$W_{d1}$ [MJ/m^3^]	$W_{d2}$ [MJ/m^3^]	$p_{max}$	$p_{E}$	$p_{f}$	$D_{f}$
SG	0.499308	0.603419	0.347815	0.511916	0.20851	0.185372	0.515869
SG1	0.505133	0.603326	0.366526	0.468787	0.194389	0.170674	0.47081
SG2	0.589216	0.705462	0.424111	**0.477499**	0.197288	**0.173662**	**0.479912**
SG3	0.05975	0.07177	0.042534	**0.489298**	0.201177	**0.177693**	**0.49224**
SG4	0.108682	0.132372	0.073482	0.54186	0.217981	0.195436	0.547126

**Table 4:** Strengths and minimum porosities for the analyzed SG concretes.

Concrete	$p_{min}$	$f_{US1}$ [MPa]	$\sigma_{ef}$ [MPa]	$f_{US2}$ [MPa]	$\sigma_{f}$ [MPa]
SG	0.0949	127.29	175.93	56.36	**66.89**
SG1	0.08	143.74	194.08	68.73	**76.26**
SG2	0.0829	151.88	206.06	71.52	**80.51**
SG3	0.087	30.66	41.87	14.14	**16.22**
SG4	0.1059	37.45	52.59	15.69	**19.4**

**Table 5:** Mechanical properties of wood and concrete at the initial moment of time $t_{0}$, and at a limit state according to rheological dynamics.

.	Initial Moment of Time ($t_{0}$)				Limit State (t=6 Years)			
	E_H_ [GPa]	µ	fc [MPa]	ρ [kg/m3]	E_H_ [GPa]	µ	fc [MPa]	ρ [kg/m3]
Wood	9.120	0.35		383.53	2.736	0.35		383.53
Concrete	7.110	0.18	6.76	1245.43	2.133	0.35	6.76	1245.43

**Table 6:** Coordinates of the measured points.

	1	2	3	4	5	6	7
Left (L)	186.71	183.29	175.92	178.04	183.27	189.97	193.52
Right (R)	194.94	187.58	188.71	190.74	199.45	206.47	214.84

## Data Availability

The research data produced in this study can be obtained by contacting the authors.
